# Health-related quality of life after risk-reducing hysterectomy (PRESCORES): a cross-sectional study

**DOI:** 10.1016/j.eclinm.2026.104083

**Published:** 2026-07-27

**Authors:** Samuel George Oxley, Milly Lake, Amanda Dibden, Tia Whenham, Xia Wei, Lea Mansour, Ashwin Kalra, Subhasheenee Ganesan, Jacqueline Sia, Michail Sideris, Caitlin Fierheller, Margarida Kurzer, Rachel Perfect-Lake, Jyotsna Pundir, Ruth Armstrong, Janos Balega, Ishita Bhatnagar, Michela Cicchetti, Cecilia Compton, Jacqueline Cook, Rosemarie Davidson, Lesa Dawson, Sadaf Ghaem-Maghami, Helen Hanson, Rachel Harrison, Rachel Hart, Alison Kraus, Fiona Lalloo, D Gareth Evans, Terri P. McVeigh, Kevin Monahan, Laura Monje-Garcia, Esther Moss, Alex Murray, Kai Ren Ong, Myfanwy Rawson, Dimitra Repana, Adam N. Rosenthal, Neil Ryan, Lyndall Sarkies, Saba Sharif, Lucy Side, Kate Simon, Tracy Smith, Joyce Solomons, Vishakha Tripathi, Amy Watford, Mark Willett, Adam Brentnall, Rosa Legood, Ranjit Manchanda, PRESCORES study team

**Affiliations:** a Wolfson Institute of Population Health, Queen Mary University of London, London EC1M 6BQ, UK; b Department of Gynaecological Oncology, Barts Health NHS Trust, Royal London Hospital, London, UK; c Department of Health Services Research and Policy, London School of Hygiene & Tropical Medicine, London WC1H 9SH, UK; d Departments of Medical Genetics and Paediatric Neurology, Cambridge University Hospitals NHS Foundation Trust, Cambridge, UK; e Pan-Birmingham Gynaecological Cancer Centre, City Hospital, Birmingham, UK; f Oxford Centre for Genomic Medicine, Oxford University Hospitals NHS Foundation Trust, Oxford, UK; g St Mark’s Hospital Centre for Familial Intestinal Cancer, London North West University Healthcare NHS Trust, London, UK; h Clinical Genetics Department, Guy’s and St Thomas’ NHS Foundation Trust, London, UK; i Sheffield Clinical Genetics Service, Sheffield Children’s NHS Foundation Trust, Sheffield, UK; j West of Scotland Genetic Service, Queen Elizabeth University Hospital, NHS Greater Glasgow and Clyde, Glasgow, UK; k Department of Obstetrics and Gynaecology, The University of British Columbia, Vancouver, Canada; l Imperial College London, London, UK; m Peninsula Clinical Genetics, Royal Devon University Healthcare NHS Foundation Trust, Exeter, UK; n Department of Clinical Genetics, Nottingham University Hospitals NHS Trust, Nottingham, UK; o Liverpool Centre for Genomic Medicine, Liverpool Women’s NHS Foundation Trust, Liverpool, UK; p Yorkshire Regional Genetics Service, Chapel Allerton Hospital, Leeds Teaching Hospitals NHS Foundation Trust, Leeds, UK; q Manchester Centre for Genomic Medicine, Manchester University Hospitals Foundation Trust, Manchester, UK; r Division of Evolution and Genomic Sciences, University of Manchester, Manchester, UK; s Cancer Genetics Unit, The Royal Marsden NHS Foundation Trust, London, UK; t Department of Gynaecological Oncology, University Hospitals of Leicester NHS Trust, Leicester, UK; u All Wales Medical Genomics Services, University Hospital of Wales, Cardiff, UK; v West Midlands Regional Genetics Service, Birmingham Women’s Hospital, Birmingham, UK; w Department of Oncology, St George’s University Hospitals NHS Foundation Trust, London, UK; x Department of Gynaecological Oncology, University College London Hospitals NHS Foundation Trust, London, UK; y Department of Gynaecology Oncology, Royal Infirmary of Edinburgh, NHS Lothian, Edinburgh, UK; z Centre for Reproductive Health, Institute of Regeneration and Repair, University of Edinburgh, Edinburgh, UK; aa North East Thames Regional Genetics Service, Great Ormond Street Hospital for Children NHS Foundation Trust, London, UK; ab Wessex Clinical Genetics Service, Princess Anne Hospital, Southampton, UK; ac Lynch Syndrome UK, West Midlands, UK; ad Department of Clinical Genetics, University Hospitals Bristol and Weston NHS Foundation Trust, Bristol, UK; ae Department of Gynaecology, East Lancashire Hospitals NHS Trust, Burnley, UK

**Keywords:** Lynch, Utilities, Risk-reducing, Hysterectomy, Quality-of-life, MMR

## Abstract

**Background:**

Understanding the impact of risk-reducing hysterectomy (RRH) on health-related quality-of-life is essential for informed decision-making around endometrial cancer prevention and has not been previously investigated. This study aimed to estimate health-related utilities in women who received RRH.

**Methods:**

PRESCORES was a multicentre cross-sectional study (ISRCTN17432105). We invited 4031 UK women ≥18 years with confirmed Lynch Syndrome, without prior gynaecological cancer, to participate between May 2023 and November 2024. The main outcome was a complete-case adjusted mean-difference in EQ-5D utility scores between participants who had undergone RRH or not, and by pre/post-menopausal age groups. Quality-of-life was measured at the time of survey completion, and retrospectively 4 months after surgery in the RRH-group. The Cancer–Worry Scale instrument was also evaluated by RRH group. Sensitivity analysis considered alternative statistical methods, multiple imputation, and restricting analysis to people 35–80 years.

**Findings:**

Analysis included 1120 participants: 529 had undergone RRH with a median age of 55 years (IQR 48–62), compared with 39 years (33–52) without RRH. The RRH-group was significantly more likely to have had cancer, physical co-morbidities, be parous and used aspirin. Adjusted mean utilities were slightly lower in the RRH than no-RRH group (difference −0.020, 95% CI −0.045 to 0.004; p = 0.11), much lower for premenopausal women (−0.041, −0.074 to −0.008), but similar for postmenopausal women (0.003, −0.036 to 0.042). Cancer-Worry-Scale scores were lower in the RRH group (mean-difference 0.48, 95% CI −0.807 to −0.157; p = 0.0037). Interpretation was robust to sensitivity analyses.

**Interpretation:**

Health-related quality-of-life in women with Lynch Syndrome is likely to be lower following RRH in younger, pre-menopausal women. This is relevant for counselling, RRH decision making, and economic evaluations of women with Lynch Syndrome, with increased endometrial cancer risk.

**Funding:**

Rosetrees Trust (CF1\100001) and Barts Charity (G-002286 and MRC0167).

**Research in context**

**Evidence before this study**

We searched PubMed, Medline & Embase from inception to January 2026, using the terms “quality of life”, “utilities”, “hysterectomy”, “risk-reduction”, “Lynch”, including synonyms and variant spellings. No previous studies have estimated the health-related quality-of-life following risk-reducing hysterectomy for endometrial cancer prevention, using EQ-5D or any other validated questionnaires. Studies which used qualitative interviews or non-validated questionnaires described a reduction in cancer worry and high satisfaction with low decision regret.

**Added value of this study**

This study is the first to report quality-of-life in women with Lynch Syndrome who have undergone risk-reducing hysterectomy and those who have not, and the first to report health utilities. It compared those who have and have not undergone risk-reducing hysterectomy to estimate the impact of surgery on quality-of-life. It recruited a large number of women with confirmed Lynch Syndrome from across the UK, and used an internationally validated and recommended tool (EQ-5D). This found that women younger than 52 years with Lynch syndrome who had risk-reducing hysterectomy report a lower quality-of-life than premenopausal women without hysterectomy, but there was no difference in older women.

**Implications of all the available evidence**

This is relevant for women with Lynch Syndrome, and may be relevant for other women at increased endometrial cancer risk, who are considering risk-reducing hysterectomy or other risk-management approaches. This information can inform counselling and shared decision-making on endometrial cancer prevention strategies, which should address not only the preventive benefit of surgery but also the potential quality-of-life trade-offs. It is also important for health economic evaluations internationally.

## Introduction

Endometrial cancer is the most common gynaecological cancer in developed countries affecting ∼3% of women,^1^ and incidence is projected to increase by 53% by 2040.^2^^,^^3^ Lynch Syndrome affects 1 in 280–370 people,^4^^,^^5^ and accounts for 3% of endometrial cancer cases,^6^ predisposing carriers to 13–49% lifetime-risk of endometrial cancer, along-with an increased risk of ovarian and colorectal cancer and other malignancies.^7^ Although only an estimated <5% of carriers have been identified,^5^ this will likely increase with the implementation of routine tumour and targeted-germline screening at endometrial and colorectal cancer diagnosis^8^ and prospective trials of unselected population genetic-testing.^9^ Beyond Lynch Syndrome, many more women may be identified at elevated endometrial cancer-risk with the advent of validated personalised endometrial cancer risk-prediction tools.^10^

For women at increased endometrial cancer-risk, risk-reducing hysterectomy (RRH) is the gold-standard preventive strategy which reduces endometrial cancer-risk, likely with ∼100% efficacy.^11^ Gynaecological surveillance with annual transvaginal ultrasound and/or endometrial biopsy may be used as an interim strategy, however there is no high-quality evidence that surveillance improves endometrial cancer outcomes.^12^^,^^13^ This is recommended to Lynch-carriers from 35 to 40 years after completing childbearing, usually with bilateral salpingo-oophorectomy (BSO) to reduce the concomitant ovarian cancer-risk.^14^^,^^15^ RRH has potential surgical complications and cessation of natural fertility.^16^ Premenopausal RRH-BSO leads to surgical menopause, for which hormone-replacement-therapy (HRT) helps treat vasomotor and other symptoms, and avoid long-term detrimental sequaelae.^17^ The decision for risk-reducing surgery is complex, guided by perceived cancer risk, perceived treatment benefit, fertility wishes, long-term health consequences, and other psychosocial factors.^18^^,^^19^ Patients must weigh the benefits of cancer prevention against the potential impact on quality-of-life (QoL).^20^ An understanding of QoL after RRH is therefore highly relevant for counselling and decision-making around RRH, and essential for health-economic evaluations of endometrial cancer prevention, which inform clinical guidelines. These require QoL ratings in the form of utilities, which represent QoL on a spectrum from ‘1’ being perfect QoL to ‘0’ being death.

No studies have reported utilities following RRH, despite its need, including for economic evaluations,^21^ and being identified as a research priority by policy-makers like National-Institute of Health-and Care-Excellence (NICE).^22^ As women with Lynch Syndrome are the major patient-group who currently undergo RRH, any direct assessment of QoL following RRH must involve such patients. Given that a randomised controlled trial of risk-reducing surgery versus control/surveillance would be unethical, and unacceptable to high-risk patients, any study must be non-randomised. A prospective study would take numerous years to recruit sufficient numbers to enable a comparison of RRH versus no RRH groups and require substantial funding, and therefore a cross-sectional retrospective study is required to provide an estimation of utilities within a reasonable timeframe.

This study aims to determine the health-related utility scores for risk-reducing hysterectomy for endometrial cancer prevention, overall and separately for pre- and post-menopausal patients.

## Methods

### Study design and participants

PRESCORES (ISRCTN17432105) was a multi-centre cross-sectional study of women with Lynch syndrome.

Eligible adult UK-women (≥18 years) were proven Lynch Syndrome pathogenic-variant carriers, who could understand written English, and had no history of endometrial, ovarian or cervical cancer (given their treatment usually precludes RRH). Participants were identified by 25 National-Health-Service (NHS) trusts across England, Scotland and Wales, with additional publicity through the patient charity Lynch syndrome UK. Participants were sent the study information pack and could consent and complete the study questionnaire electronically or on paper.^23^^,^^24^

### Ethics

This study was approved by the Surrey Research Ethics Committee on 05/12/2022 (22/PR/1167).

The questionnaire and other patient-facing materials were peer-reviewed by patient representatives including from the Lynch Syndrome UK charity (https://www.lynch-syndrome-uk.org/). Lynch Syndrome UK supported increasing awareness of the research, including through social-media channels, and were represented on the study management group and manuscript co-authorship.

### Procedures

The study questionnaire included the EQ-5D-5L questionnaire (NICE recommended^25^) to measure QoL at time of survey completion.^26^ Patient demographics, medical and surgical history, visual-analogue scale (VAS), and the Cancer–Worry Scale were also captured.^27^ A retrospective EQ-5D for QoL of patients 4 months after RRH was also recorded for those who had RRH.

### Outcomes

The primary endpoint was the adjusted mean-difference in *current* utilities between those who had RRH versus not overall, and secondarily in subgroups by (a) women <51 years in the RRH group versus premenopausal women in no-RRH group; (b) women ≥51 years in the RRH group versus postmenopausal women in no-RRH group.

An exploratory outcome was the age-standardised difference in utilities between women with Lynch Syndrome and the general female-population in England.

### Statistics

Sample size was planned by considering the population of 5112 identified female Lynch Syndrome carriers in England,^28^ of which around 30% have undergone RRH^29^; around 1500 women. We aimed to recruit 750 women with Lynch Syndrome overall (range 300–1200) including 250 women who had RRH (range 100–400). This was consistent with previous questionnaire studies in women at inheritable risk of gynaecological cancers, and was expected to include a representation from all types of carriers,^30^ and to allow for informative post-hoc exploratory analysis.

Utilities were calculated by mapping between EQ-5D-5L and 3L using the NICE-recommended UK value-set.^25^^,^^26^ Patients were excluded if responses for the following variables were incomplete or uncertain: age, RRH-status, and EQ-5D domains. Participants were separated into “RRH” and “no-RRH” groups based on self-reported RRH ± BSO status. Patients who reported hysterectomy for cancer treatment or benign gynaecological disease, and BSO (without hysterectomy) were excluded. Patients who underwent unilateral salpingo-oophorectomy (without hysterectomy) were classified as no-RRH.

Menopausal status of ‘no-RRH group’ women was defined in relation to menstrual periods; patients reporting no periods in the previous 12-months were classed as postmenopausal, unless this was due to contraception or other hormonal medication. Patients reporting ≥1 period in the last 12-months were classed as premenopausal, unless they were ≥56 years.^31^ Patients in the RRH-group were compared against pre/postmenopausal patients in the no-RRH group based on current age under/over 51 years (mean-age of menopause in UK women^31^), respectively. This enabled a comparison of patients who underwent RRH against an equivalent cohort of participants who have not, to estimate the impact of RRH on current QoL. For the sensitivity analysis, patients in the RRH-group were compared based on age at RRH under or over 51 years.

A physical health condition was defined as any physical health condition other than cancer. A mental health condition was defined as any such condition, including anxiety, depression or other. Categorical variables were dichotomised post-hoc for the regression-analysis, including marital status (married/cohabiting versus other), household income (>£40,000 versus <£40,000, given median household-income of £38,000^32^), and education level (university/college versus secondary/high-school).

Primarily, complete-case analysis was performed to evaluate the adjusted mean difference in utilities between RRH groups using linear regression overall, and by pre/post menopause. This used Ordinary-Least-Squares (OLS) regression, using the normal approximation of the bootstrap standard error for confidence intervals.^33^, ^34^, ^35^, ^36^ Other analysis tested independence between categorical variables by RRH group using Chi-squared, or Fisher’s-exact test if the expected cell counts were less than five for any cell. For continuous variables, T-tests were used if the data appeared to be normally distributed, and Mann-Whitney-U tests if the data did not appear normally distributed, based on visual inspection. Correlation between total Cancer-Worry-Scale scores and utilities used Spearman’s-coefficient. Due to the observational nature of this study, adjustment for confounders is vital for interpretation. We focused on factors that were associated with utilities in the no-RRH group and judged unlikely to be substantially caused by RRH. Since many potential variables were captured in the study, we opted to use a partly data-driven approach to choose those most strongly associated with utilities in the no-RRH group, using a Least-Absolute-Shrinkage and Selection-Operator (LASSO) linear-regression model. The impact of time since RRH to survey completion on utilities was evaluated within the RRH-group only, and in separate analysis stratifying the RRH-group by years since RRH: 0–2 years, 3–5 years, ≥6 years, comparing each against the no-RRH group.

Exploratory analysis compared utilities from the PRESCORES sample with general female-population values for England, using a recent Health-Survey for England analysis based on 8094 women.^37^ EQ-5D utilities from PRESCORES participants were age-standardised against the reference population^37^ using direct standardisation, and the adjusted difference in mean-utilities between the PRESCORES and reference populations was assessed by OLS. We performed further subgroup-analysis for participants with/without a personal cancer-history.

Several sensitivity analyses to evaluate robustness were conducted: 1) The primary analysis was repeated for people 35–80 years (RRH is only recommended for those ≥35 years); 2) The impact of missing data was evaluating using multiple imputation based using multiple imputation by chained equations, drawing an imputed sample in each bootstrap re-sample^38^; 3) An alternative model was used for primary analysis in people aged 35–80 years old, embedding propensity score weighting within bootstrap resampling, in a complete-case or multiple imputation framework. This used the same adjustment factors as the primary analysis. Here the marginal average treatment effect was estimated with inverse probability weighting applied to trimmed (99% percentile) weights, and through a doubly-robust approach^39^; 4) An unadjusted sensitivity-analysis was conducted for the subset of patients who underwent RRH without BSO; 5) A further sensitivity analysis aimed to determine the adjusted mean-difference in utilities 4-months *after RRH* between RRH and no-RRH groups, comparing utilities 4-months *after RRH* in the RRH-group to *current* utilities in ‘no-RRH group’ women. The three additional retrospective analyses were: 1) Utilities 4-months after RRH in the RRH group versus current utilities in no-RRH group; 2) Utilities 4-months after RRH in women who underwent RRH < 51 years versus current utilities of premenopausal women in no-RRH group; 3) Utilities 4-months after RRH in women who underwent RRH ≥ 51 years versus current utilities of postmenopausal women in no-RRH group.

All statistical tests were two-sided, at 5% significance level, without adjustments for multiple testing. Data cleaning were undertaken using SPSS version 29, with primary, secondary and exploratory analyses undertaken using Stata version 18, and some sensitivity analyses using R version 4.4.2, with R packages MatchIt version 4.7.2 for propensity score matching and WeightIt for inverse probability weighting.^40^

### Role of funding source

This study was funded by the Rosetrees Trust (CF1\100001 5) and Barts Charity (G-002286 and MRC0167). Funders had no role in the design, data collection, data analysis, interpretation or writing of the report.

## Results

**Fig. 1:**
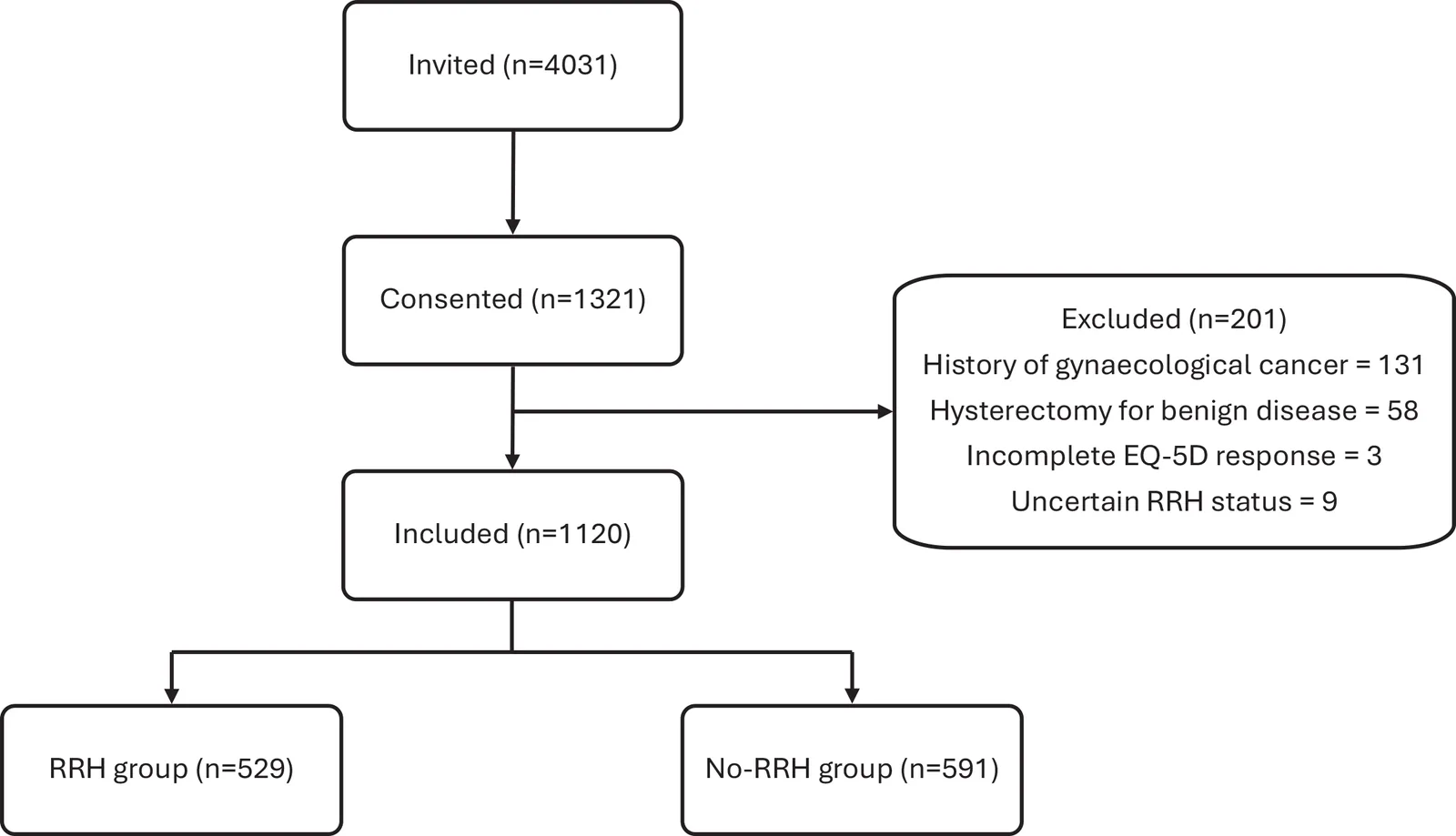
Study flow diagram.

In total, 4031 patients with Lynch Syndrome were directly invited to participate from 25 NHS trusts across England, Scotland and Wales (Appendix Fig. S1) between May 2023 and November 2024. It is not possible to know how many additional patients saw Lynch Syndrome UK publicity. Overall, 1321 participants provided informed consent to participate in the study and returned questionnaires. After excluding 201 patients, 1120 participants were included for analysis (Fig. 1).

The clinical and demographic characteristics of included participants are summarised in Table 1. Of the 529 participants who underwent RRH, 500 had RRH-BSO and 29 had RRH without BSO. RRH and no-RRH groups differed significantly with respect to age and some clinical and demographic factors.

**Table 1:** Characteristics of included participants, provided as numbers (% of column total).

	RRH	no-RRH	Overall	p-value
N	529	591	1120	
Mean age, yrs [SD]	54.9 [9.8]	42.9 [14.2]	48.6 [13.7]	<0.0001^a^
Median age, yrs [IQR, range]	55 [48–62, 32–82]	39 [33–52, 18–87]	49 [38–59, 18–87]	
Lynch Syndrome genes				
*MLH1*	120 (22.7)	127 (21.5)	247 (22.1)	<0.0001^a^
*MSH2*	150 (28.4)	137 (23.2)	287 (25.6)	
*MHS6*	118 (22.3)	97 (16.4)	215 (19.2)	
*PMS2*	48 (9.1)	90 (15.2)	138 (12.3)	
*EPCAM*	2 (0.4)	8 (1.4)	10 (0.9)	
Not sure/missing	91 (17.2)	132 (22.3)	223 (19.9)	
Mean age at RRH, years (SD)	47.4 (8.9)	–	–	–
Median years since RRH (IQR)	6 (2–11)	–	–	–
Menopausal status/age				
Premenopausal	189 (35.7)	433 (73.3)	–	<0.0001^a^
Postmenopausal	340 (64.3)	158 (26.7)	–	
HRT ever user				
No	181 (34.2)	511 (86.5)	692 (61.8)	<0.0001^a^
Yes	336 (63.5)	67 (11.3)	403 (36.0)	
Not sure	1 (0.19)	5 (0.85)	6 (0.54)	
Missing	11 (2.1)	8 (1.4)	19 (1.7)	
HRT current user				
No	266 (51.5)	532 (92.0)	798 (72.9)	<0.0001^a^
Yes	251 (48.6)	46 (8.0)	297 (27.1)	
Missing	12 (2.3)	13 (2.2)	25 (2.2)	
Personal cancer history				
No	326 (61.6)	445 (75.3)	771 (68.8)	<0.0001^a^
Yes	202 (38.2)	145 (24.5)	347 (31.0)	
Missing	1 (0.19)	1 (0.17)	2 (0.18)	
Cancer type^b^:				
Bowel	144 (71.3)	108 (74.5)	252 (72.6)	
Breast	36 (17.8)	28 (19.3)	64 (18.4)	
Other	43 (21.3)	22 (15.2)	65 (18.7)	
Number of physical comorbidities				
0	257 (48.6)	334 (56.5)	591 (52.8)	0.0061
1	152 (28.7)	163 (27.6)	315 (28.1)	
2+	120 (22.7)	94 (15.9)	214 (19.1)	
Mental health condition^b^				
No	386 (73.0)	421 (71.3)	807 (72.1)	0.32
Yes	136 (25.7)	163 (27.6)	299 (26.7)	
Missing	7 (1.3)	7 (1.2)	14 (1.3)	
Anxiety	110 (80.9)	136 (83.4)	246 (82.3)	
Depression	67 (49.3)	74 (45.4)	141 (47.2)	
Other	9 (6.6)	28 (17.2)	37 (12.4)	
Have children				
No	65 (12.3)	215 (36.4)	280 (25.0)	<0.0001^a^
Yes	454 (85.8)	372 (63.0)	826 (73.8)	
Missing	10 (1.9)	4 (0.68)	14 (1.3)	
Aspirin use				
No	321 (60.7)	416 (70.4)	737 (65.8)	<0.0001^a^
Yes	195 (36.9)	166 (28.1)	361 (32.2)	
Not sure	1 (0.2)	3 (0.51)	4 (0.36)	
Missing	12 (2.3)	6 (1.0)	18 (1.6)	
Attend colorectal screening				
No	26 (4.9)	89 (15.1)	115 (10.3)	<0.0001^a^
Yes	491 (92.8)	491 (83.1)	982 (87.7)	
Not sure	1 (0.19)	6 (1.0)	7 (0.62)	
Missing	11 (2.1)	5 (0.85)	16 (1.4)	
Attend endometrial cancer screening				
No	–	393 (66.5)	–	–
Yes	–	162 (27.4)	–	
Not sure	–	31 (5.3)	–	
Missing		5 (0.85)		
Family history of cancer				
No	13 (2.5)	13 (2.2)	26 (2.3)	0.77
Yes	503 (95.1)	565 (95.6)	1068 (95.4)	
Not sure	3 (0.57)	8 (1.4)	11 (0.98)	
Missing	10 (1.9)	5 (0.85)	15 (1.3)	
Ethnicity				
White	483 (91.3)	537 (90.9)	1020 (91.1)	0.088
Asian	12 (2.3)	19 (3.2)	31 (2.8)	
Black	7 (1.3)	6 (1.0)	13 (1.2)	
Other	7 (1.3)	21 (3.6)	28 (2.5)	
Missing	20 (3.8)	8 (1.4)	28 (2.5)	
Marital status (%)				
Single	40 (7.6)	116 (19.6)	156 (13.9)	<0.0001^a^
Married/Cohabiting	395 (74.7)	407 (68.9)	802 (71.6)	
Divorced/Separated	51 (9.6)	37 (6.3)	88 (7.9)	
Widowed	19 (3.6)	17 (2.9)	36 (3.2)	
Missing	24 (4.5)	14 (2.4)	38 (3.4)	
Education level				
No formal	20 (3.8)	26 (4.4)	46 (4.1)	<0.0001^a^
GCSE/Equivalent	152 (28.7)	98 (16.6)	250 (22.3)	
A-level/Equivalent	93 (17.6)	93 (15.7)	186 (16.6)	
Higher Education	242 (45.8)	359 (60.7)	601 (53.7)	
Missing	22 (4.2)	15 (2.5)	37 (3.3)	
Income				
Less than £10,000	20 (3.8)	29 (4.9)	49 (4.4)	0.17
£10,000–£19,999	73 (13.8)	58 (9.8)	131 (11.7)	
£20,000–£29,999	72 (13.6)	82 (13.9)	154 (13.8)	
£30,000–£39,999	57 (10.8)	83 (14.0)	140 (12.5)	
£40,000–£49,999	68 (12.9)	72 (12.2)	140 (12.5)	
£50,000 or more	190 (35.9)	236 (39.9)	426 (38.0)	
Missing	49 (9.3)	31 (5.3)	80 (7.1)	

RRH—risk-reducing hysterectomy, IQR—interquartile range; HRT—Hormone replacement therapy. p-value indicates the result of a Chi squared/Fisher’s exact test or Mann-Whitey U test to compare RRH and no-RRH groups and are calculated using known values only.

a Indicates statistical significance at 0.05.

b Values may sum to over 100% due to multiple non-exclusive comorbidities/variable options.

**Fig. 2:**
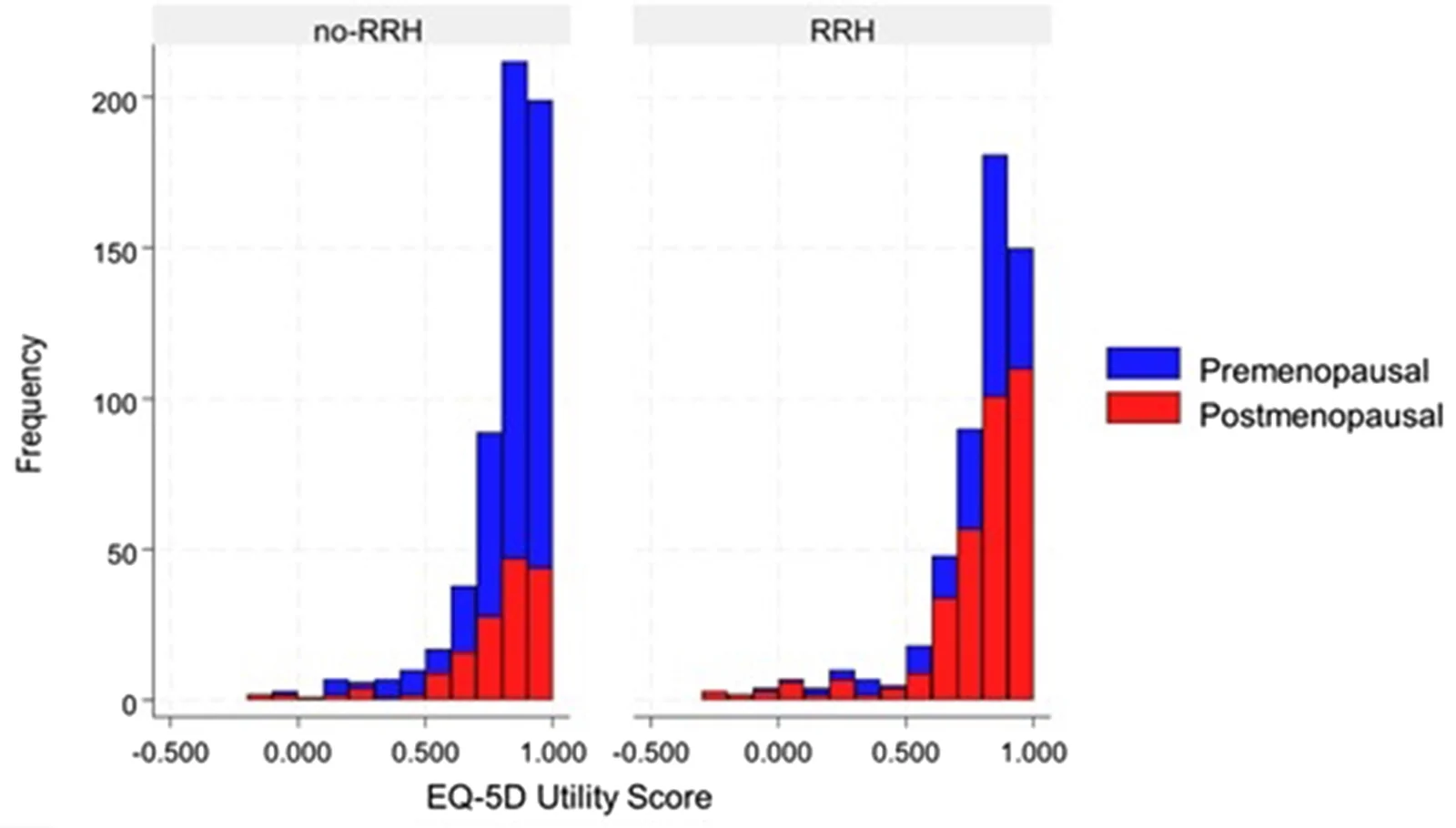
Unadjusted utilities of “Risk-reducing hysterectomy (RRH)” and “No-RRH” groups.

Responses to the five EQ-5D domains are reported in Appendix Table S1. No-RRH group patients were less-likely to have problems with mobility (p < 0.0001), doing usual activities (p = 0.0010), and pain/discomfort (p = 0.013), compared to RRH-group patients. The overall unadjusted mean-utilities within the whole study population was 0.812 (SD 0.207), 0.793 (0.225) within the RRH-group and 0.828 (0.189) in the no-RRH-group. These results are shown in Fig. 2, including premenopausal/postmenopausal-age comparison groups. See Appendix Table S2 for the full dataset.

The factors associated with utilities in the no-RRH-group and used for adjustment were: presence of any physical health condition (−0.091, 95% CI −0.117 to −0.065; p < 0.0001), mental health condition (−0.165, −0.203 to −0.128; p < 0.0001), household income ≥£40,000/year (0.042, 0.017 to 0.068; p = 0.0013), and a personal cancer history (−0.053, −0.086 to −0.021; p = 0.0013). Age was not significantly associated with utilities after adjustment for these factors but was also included as an adjustment because it is still an important confounder that is associated with EQ-5D, and differed substantially between groups.

Primary analysis revealed slightly lower utilities in the RRH group (adjusted mean difference −0.020, 95% CI −0.045 to 0.004; p = 0.11; complete-case n = 1032, n = 478 RRH). Utilities were significantly lower in the premenopausal RRH group (adjusted mean −0.041, −0.074 to −0.008; p = 0.014, n = 584) but similar (0.003, −0.036 to 0.042; p = 0.88, n = 448) in the postmenopausal group (Table 2).

**Table 2:** Adjusted mean-difference in current utilities between “Risk-reducing hysterectomy (RRH)” and “no-RRH” groups, overall, and by current age in the RRH group (</≥51 years), and menopausal status in the no-RRH group.

	n RRH/no-RRH	Adjusted mean difference in utilities	Bootstrap SE	95% CI	p-value
RRH versus no-RRH^a^	478/554	−0.020	0.013	−0.045, 0.004	0.11
RRH (<51 yrs) versus no-RRH (premenopausal)^a^	171/413	−0.041	0.017	−0.074, −0.008	0.014
RRH (≥51 yrs) versus no-RRH (postmenopausal)^a^	307/141	0.003	0.020	−0.036, 0.042	0.88

RRH—risk-reducing hysterectomy; SE—standard error; CI—confidence interval; Premen—premenopausal; Postmen—postmenopausal.

a Adjusted for physical health, mental health, income, cancer history and age.

Further exploratory analysis found little evidence of an association between current HRT-use and utilities in the RRH-group (p = 0.29). Sensitivity analysis restricting the age range to 35–80 years (n = 918, n = 520 RRH) and using other statistical modelling methods suggested robust findings (Appendix Table S3). Sensitivity analysis of RRH without BSO (n = 29) versus all no-RRH showed a smaller overall difference in mean utilities (−0.111, 95% CI −0.227 to −0.005; p = 0.061), and in the postmenopausal-age comparison subgroup (−0.284, −0.498 to −0.070; p = 0.0092) (Appendix Table S4).

Amongst those with RRH there was little evidence of an association between utilities and time since RRH (p = 0.50). When stratifying by years of follow-up between RRH < 51 years and premenopausal no-RRH women, there was a small difference at 0–2 years (−0.049, 95% CI −0.086 to −0.013; p = 0.0075) but not much at 3–5 years of follow-up (−0.041, −0.093 to 0.010; p = 0.12) or ≥6 years (−0.070, −0.137 to −0.002; p = 0.043). There was little evidence of difference between RRH ≥ 51 years and postmenopausal no-RRH women in any follow-up interval (Appendix Table S5).

Of RRH-group participants, 490 completed the retrospective EQ-5D on their health and QoL 4 months after RRH (missing n = 39). The mean utility was 0.79 (SD 0.232), which was the same as the utility within the RRH-group. As in the main analysis, the regression-analysis found an adjusted difference of −0.026 (95% CI −0.054 to 0.001; p = 0.063) in utilities between RRH and no-RRH groups overall, with an adjusted difference of −0.080 (−0.118 to −0.043; p < 0.0001) in the premenopausal group (Appendix Table S6). These results should be interpreted with caution given their susceptibility to recall bias.

Cancer-Worry-Scale total scores were lower in RRH-group compared to no-RRH-group women (mean difference 0.48, 95% CI −0.807 to −0.157; p = 0.0037; Appendix Table S7), and this did not differ by mismatch repair gene. Lower total-scores were associated with higher utilities across all patients (−4.25, p < 0.01; Appendix Fig. S2).

**Fig. 3:**
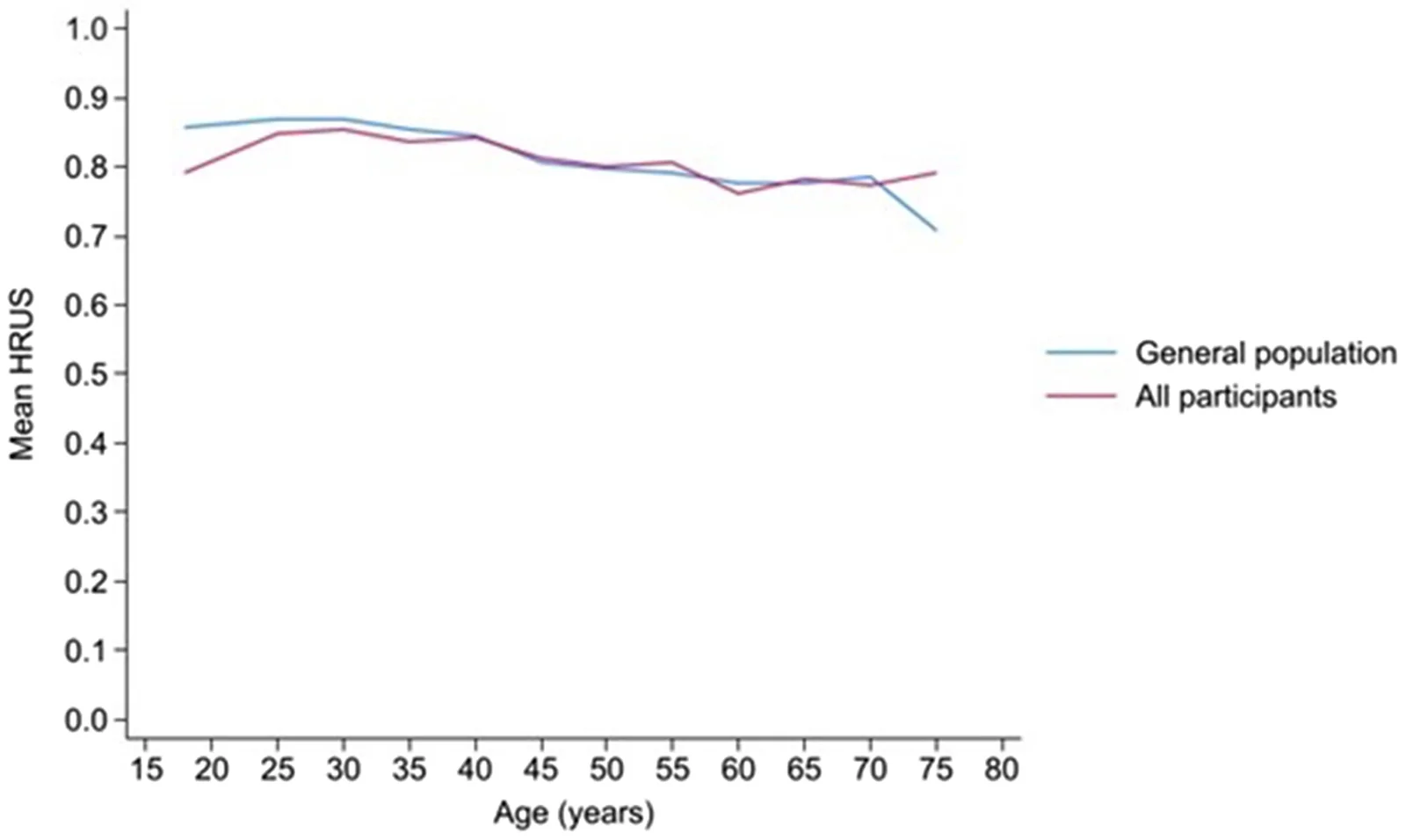
Utilities of all study participants versus the English general female population.

There was very little difference in the age-standardised utility scores between all Lynch Syndrome carriers and the England general population (0.001, 95% CI −0.034 to 0.036; p = 0.96; Fig. 3). This was seen for those with and without a personal cancer history (Appendix Table S8).

## Discussion

This study is the first to report health-related QoL after RRH and of women with Lynch Syndrome using EQ-5D, finding that RRH is associated with a decrease in utilities for predominantly premenopausal women (−0.041, 95% CI −0.074 to −0.008), but not post-menopausal women. Increasing time since RRH was not associated with a difference in QoL, although a small difference was seen in subgroup analyses after premenopausal-age RRH.

A small difference in QoL was found between RRH without BSO and no-RRH patients. However, very few patients were included in this sensitivity analysis, making inference difficult. A retrospective comparison of utilities 4 months after RRH found a similar −0.026 overall difference following RRH versus no-RRH women (95% CI −0.054 to0.001), although with a greater −0.080 difference following premenopausal-age RRH (95% CI −0.118 to −0.043), and similarly little difference after postmenopausal-age RRH.

RRH was associated with lower cancer-worry, and cancer-worry was inversely correlated with QoL. QoL of women with Lynch Syndrome was similar to the reference England female general population.

Our analysis provides an estimate of the distribution of utilities in Lynch Syndrome carriers. The mean-difference in utilities for pre-menopausal women who have undergone RRH, and the lack of difference for post-menopausal women, is relevant for clinical counselling, RRH decision-making and health-economic evaluations. The study used a validated questionnaire (EQ-5D) in patients with experience of RRH to determine utilities, as-per the NICE “reference-case”.^25^ It recruited a large, representative sample of identified women with Lynch Syndrome from across the UK; approximately 26% of all known Lynch carriers.^28^ However, whilst the study population may be representative of identified carriers, given only an estimated <5% of carriers are identified,^5^ this may not be representative of all UK women with Lynch Syndrome. Nevertheless, this is unlikely to affect the main outcome of adjusted mean-difference in utilities between RRH and no-RRH groups.

The cross-sectional and retrospective nature of this questionnaire-study has limitations, which include potential biases including selection-bias.^41^ Due to the non-randomised cross-sectional nature of the study, there were significant differences in baseline characteristics between the RRH and no-RRH groups, although the regression-analysis was stratified by menopausal status, provided some adjustment for comorbidities and other variables, and several sensitivity analyses were conducted. All information was self-reported, although steps were taken to remove unreliable data, such as excluding participants unsure of their hysterectomy status. We asked participants for a personal history of cancer and current cancer treatments, but do not have detailed information of cancer treatment history. Therefore, our analysis cannot account for the likely substantial heterogeneity in prior cancer treatment burden, which may introduce residual confounding between RRH and lower current utilities. Whilst our questionnaire asked about HRT duration and type, we did not capture granular data on dose or adequacy of symptom control. The absence of a menopause-specific quality-of-life instrument limits our ability to attribute the observed QoL difference to specific menopausal symptoms versus other surgical sequelae. EQ-5D captures QoL “today” and thus is not susceptible to recall bias for the primary analyses. However, this is a potential concern for retrospective questionnaire questions, particularly for the retrospective EQ-5D elicited for 4 months after RRH, and caution is required while interpreting this result. The sensitivity analysis of 29 RRH-alone (no-BSO) patients is underpowered, even more with premenopausal/postmenopausal-age subgroups, and hence, caution should be exercised when interpreting these results.

This study provides self-reported clinical and demographic details of a large population of women with Lynch Syndrome which allows comparison with Lynch Syndrome registries in England and internationally. The study population showed a similar MMR-gene distribution to the English National Lynch Syndrome Registry, although the present study has more detailed results on ethnicity, medical history and demographics.^28^ The mean-age of RRH of 48.6 years is similar to findings from the Prospective Lynch Syndrome Database with a mean-age of 45.4–53.3 years.^29^ Uptake of aspirin for medical prevention (36%) is similar to that reported in a US study (37.5%),^42^ and suggests the potential for improvement. The 88% uptake of colorectal screening is lower than reported in a regional survey of Lynch Syndrome carriers (97%),^43^ which may better reflect national levels given the lack of a national screening service during the study period. The no-RRH group differed from the RRH-group including being younger, less likely to have a personal cancer history, have children, take aspirin for medical prevention or attend colorectal cancer screening. This reflects well-understood motivations for undergoing risk-reducing surgery including a personal cancer history and completion of family.^44^^,^^45^ It may be important to ensure that Lynch Syndrome carriers who decline/postpone RRH still engage with other cancer risk-reducing interventions including aspirin if appropriate.

These findings demonstrate that women currently aged under 51 years who have undergone RRH report lower QoL than premenopausal women who have not, whilst no difference is seen in the older age group. This suggests that any QoL impact after RRH is predominantly due to menopausal symptoms arising from premenopausal BSO, particularly as they are supported by pre-specified sensitivity analyses: categorising participants by age at RRH (rather than current age), and the retrospective analysis at 4 months after RRH demonstrating a greater negative impact in women who underwent RRH at a premenopausal age, when symptoms of surgical menopause would be felt acutely.

Whilst the overall effect size of −0.02 is lower than the most commonly cited minimally-important clinical difference for EQ-5D of 0.03–0.07,^46^ the premenopausal-specific effect size of −0.041 falls within this range, and is clinically relevant for an otherwise healthy patient considering risk-reduction. The estimated size of premenopausal QoL impact was similar to predicted from vignette studies of RRSO in *BRCA1/2*-carriers reporting a utility of 0.95 (equivalent to a 0.05 disutility),^47^ used for most economic-evaluations of ovarian cancer prevention.^48^

For premenopausal carriers considering RRH, these findings support counselling about the potential modest impact of surgery on QoL. Decision making should consider the preventive benefit of surgery and also the potential QoL trade-offs. Where appropriate, the option of deferring RRH until closer to natural menopause may mitigate this QoL impact, consistent with recommendations for carriers of lower-penetrance genes such as PMS2.^14^ There was however no significant association between HRT use and utilities in the RRH-group; this may be due to limited sensitivity of EQ-5D specifically for this issue and/or self-selection effects in who receives HRT. Without data on menopause-specific QoL it is difficult to draw further conclusions, including on whether optimal HRT management would partially mitigate this observed QoL impact, and further studies would be required to confirm this.

The small reduction in cancer-worry in the RRH-group is reassuring, which inversely correlates with overall QoL. The lack of a more substantial reduction may reflect the ongoing elevated lifetime-risk of colorectal cancer and other malignancies, which are only partially mitigated with medical prevention and screening.^49^ Baseline cancer-worry may be increased in Lynch Syndrome carriers given the high (97.6%) prevalence of a family-history of cancer, and Amsterdam Criteria (family-history of cancer in ≥3 relatives linked by a first-degree relationship^50^), used historically to determine eligibility for Lynch Syndrome testing.

Whilst EQ-5D is a well-validated generic-QoL tool which enables comparison across multiple health states and is recommended for economic-evaluations,^25^ it lacks questions directly relating to menopausal QoL, urinary or sexual-functioning. It may therefore underestimate the impact of condition-specific symptoms resulting from RRH. Future exploration of these symptoms in this population may be valuable.

The results of this study are relevant for counselling patients with Lynch Syndrome and for economic evaluations of patients (undergoing any intervention) along with RRH for endometrial cancer-prevention for indications beyond Lynch Syndrome. Many more women may in future be identified at moderately increased endometrial cancer-risk from personalised risk-prediction tools, including from obesity, family-history, and other clinical factors.^10^ For premenopausal women particularly, separating the QoL impact of RRH from RRH-BSO (challenging in this study) would be helpful. It may be of greater importance in countries where RRH without BSO is more commonly practiced.^29^ Further studies are required to isolate this, including through alternative study designs such as valuations of vignettes.^25^^,^^51^

RRH is associated with a small reduction in generic QoL, particularly for premenopausal-age women with Lynch Syndrome, whilst it appears not to have an impact in postmenopausal-age women. RRH is associated with a small reduction in cancer-worry. Overall, women with Lynch Syndrome are likely to have similar overall QoL to the general population. These results are highly relevant for patients at increased endometrial cancer-risk from Lynch Syndrome. This may inform future counselling of patients which should address not only the preventive benefit of surgery but also the potential QoL trade-offs. Our findings are also relevant for associated health-economic evaluations.

## Contributors

Drs Manchanda, Legood, Oxley, Dibden, and Brentnall had full access to all the data in the study and take responsibility for the integrity of the data and the accuracy of the data analysis. Prof Manchanda is senior author and overall guarantor of the work, and responsible for the decision to submit the manuscript.

Oxley, Dibden, Brentnall, and Manchanda verified the study data.

Concept and design: Manchanda, Legood, Oxley, Brentnall.

Patient Recruitment, data acquisition: Oxley, Lake, Whenham, Kurzer, Perfect-Lake, Smith, Pundir, Armstrong, Balega, Bhatnagar, Cicchetti, Compton, Cook, Davidson, Dawson, Ghaem-Maghami, Hanson, Harrison, Hart, Kraus, Lalloo, McVeigh, Monahan, Monje-Garcia, Moss, Murray, Ong, Rawson, Repana, Rosenthal, Ryan, Sarkies, Sharif, Side, Simon, Solomons, Tripathi, Watford, Willett, Manchanda.

Data analysis, and/or interpretation: Oxley, Dibden, Lake, Whenham, Mansour, Brentnall, Legood, Manchanda.

Initial drafting of the manuscript: Oxley, Dibden, Legood, Manchanda.

Critical review of the manuscript for important intellectual content: Oxley, Dibden, Mansour, Fierheller, Kalra, Sia, Ganesan, Sideris, Wei, Pundir, Armstrong, Balega, Bhatnagar, Cicchetti, Compton, Cook, Davidson, Dawson, Evans, Ghaem-Maghami, Hanson, Harrison, Hart, Kraus, Lalloo, McVeigh, Monahan, Monje-Garcia, Moss, Murray, Ong, Rawson, Repana, Rosenthal, Ryan, Sarkies, Sharif, Side, Simon, Solomons, Tripathi, Watford, Willett, Brentnall, Legood, Manchanda.

Statistical analysis: Oxley, Dibden, Brentnall, Legood, Manchanda.

Obtained funding: Manchanda.

Administrative, technical, or material support: Oxley, Whenham, Lake, Dibden, Fierheller, Kalra, Sia, Ganesan, Sideris, Armstrong, Balega, Bhatnagar, Cicchetti, Compton, Cook, Davidson, Dawson, Ghaem-Maghami, Hanson, Harrison, Hart, Kraus, Lalloo, McVeigh, Monahan, Monje-Garcia, Moss, Murray, Ren Ong, Rawson, Repana, Rosenthal, Ryan, Sarkies, Sharif, Side, Simon, Solomons, Tripathi, Watford, Willett, Legood, Manchanda.

Supervision: Manchanda, Legood, Brentnall.

All authors have read and approved the final version of the manuscript.

## Data sharing statement

Data from this study will be shared upon reasonable request from the corresponding author (RM).

## Declaration of interests

RM declares receiving grants from Yorkshire Cancer Research, GSK, NHS England, Barts Charity, and the NHS Innovation Accelerator; receiving speaking fees from GSK, MSD; and receiving personal fees for serving on the advisory boards for EGL and AstraZeneca outside the submitted work. AK declares funding from Barts Charity. CF declares fundings from Wellbeing of Women and Peaches Womb Cancer Trust. SGM, KM, JB and MW declare speaking fees from GSK, and MW speaking fees from MSD. KM declares funding from 40 tude Curing Cancer Charity and Cancer Research UK, and service on the advisory board for the charities Bowel Cancer UK and Lynch Syndrome UK. EM declares funding from Sarcoma UK, NIHR, Hope Against Cancer, The Eve Appeal, Leicester, Leicestershire and Rutland Integrated Care Board, the North East London Cancer Alliance, and speaking fees from GSK. AM declares speaking fees from Astra Zeneca and Novartis. AB declares funding from NIHR, Cancer Research UK, Breast Cancer Now, MRC, Prostate Cancer UK, Barts Charity, Cepheid, Unicancer France, Royalties from Cancer Research UK, consulting fees from Median Technologies, Kings College London Talent Bank, participation on a data monitoring committee at Imperial College London, and other interests relating to work as a statistician on trials relating to cancer screening and prevention. ANR was supported by the NIHR Biomedical Research Centre at University College London Hospitals National Health Service Foundation Trust and University College London, has worked as an unpaid member of a data safety monitoring board, and is the director of Smart Medics ltd. NR declares Funding from the Scottish Government, Eve Appeal and North West Cancer Trust and The Melville Trust, European Union and Cancer Research UK.

## Appendix A Supplementary data

Appendix - Questionnaire
