# Supplementary material for: Health-related quality of life after risk-reducing hysterectomy (PRESCORES): a cross-sectional study
Source: eClinicalMedicine. 2026 Jul 27;98:104083. doi: 10.1016/j.eclinm.2026.104083 (PMC13523832; doi:10.1016/j.eclinm.2026.104083)
Supplement: Supplementary file 1 [file mmc1.pdf]

Survey on the quality of life after surgery to prevent womb (endometrial) cancer: part of the PRESCORES study

This survey has 3 sections:

1. Rating your own quality of life, using a simple scale and 5 questions.
2. Imagining you are another person described in a scenario who has had an operation, and rating their quality of life (using the same scale as section 1).
3. Questions about your own health and background.

Thank you for participating in this research which will help contribute to our work on the prevention of womb (endometrial) cancer.

Section 1: Your own quality of life

Please rate **your own** health and quality of life to reflect **how you feel today**. This can be important in understanding how you view the quality of life of others. It will also allow you to become familiar with the rating scale and questions used in section 2.

Your Health TODAY

This task is about rating how good or bad your health is TODAY

This scale is numbered from 0 to 100.

100 means the best health you can imagine.
0 means the worst health you can imagine.

Please **mark an X** on the scale to indicate how your health is TODAY.

Now, write the number you marked on the scale in the box below.

YOUR HEALTH TODAY = [ ]

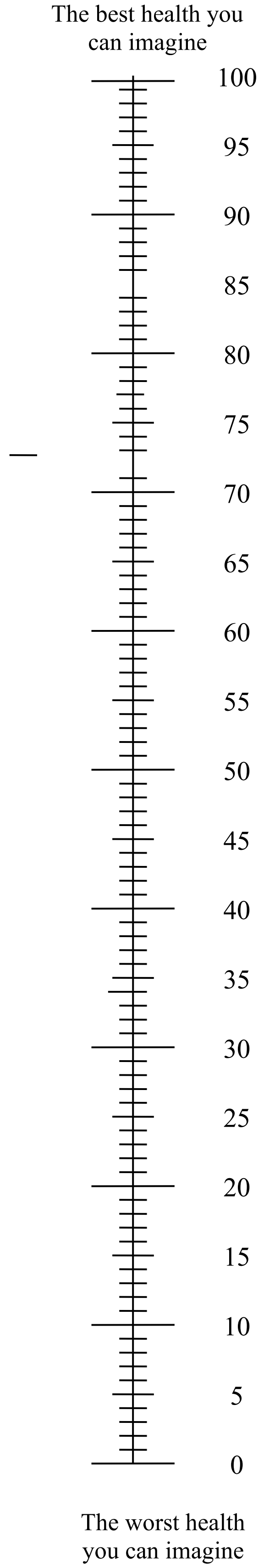

Your Quality of Life TODAY

Under each heading, please tick the <u>ONE</u> box that best describes your health TODAY.

MOBILITY

I have no problems in walking about ❑

I have slight problems in walking about ❑

I have moderate problems in walking about ❑

I have severe problems in walking about ❑

I am unable to walk about ❑

SELF-CARE

I have no problems washing or dressing myself ❑

I have slight problems washing or dressing myself ❑

I have moderate problems washing or dressing myself ❑

I have severe problems washing or dressing myself ❑

I am unable to wash or dress myself ❑

USUAL ACTIVITIES *(e.g. work, study, housework, family or leisure activities)*

I have no problems doing my usual activities ❑

I have slight problems doing my usual activities ❑

I have moderate problems doing my usual activities ❑

I have severe problems doing my usual activities ❑

I am unable to do my usual activities ❑

PAIN / DISCOMFORT

I have no pain or discomfort ❑

I have slight pain or discomfort ❑

I have moderate pain or discomfort ❑

I have severe pain or discomfort ❑

I have extreme pain or discomfort ❑

ANXIETY / DEPRESSION

I am not anxious or depressed ❑

I am slightly anxious or depressed ❑

I am moderately anxious or depressed ❑

I am severely anxious or depressed ❑

I am extremely anxious or depressed ❑

Section 2: Quality of life of another person described in the scenario

We will now ask you to read a description of another person's health.
The aim of this study is to understand how you would rate the quality of life of the person described.

Please carefully imagine that you are the person described, think how you would feel and what your life would be like. After reading the description, we will ask you to complete the same health and quality of life assessment as before, as though you were the person in the scenario. Please answer as best as you can, from the information provided.

[Randomise to either scenario A/ B/ C/ D]

[Scenario A]

About you

- You are 40 years old. You have children and have completed your family.
- You have no medical conditions affecting your general health.
- You have been told you carry a genetic alteration, which increases your risk of womb cancer in future.
- You have had yearly screening for womb cancer. This included an ultrasound scan and a small camera inserted into the womb to take a sample from the lining of the womb.
- You discussed your options to prevent womb cancer with a specialist. You decided to have an operation to remove your womb to prevent womb cancer.

Treatment

- **1 month** ago you had an operation to **remove your womb**. You did **not** have your ovaries removed.
- The operation went well, and you went home the next day.

Symptoms

- You have 4 small scars on your abdomen, each 1-2cm. These are healing well and are not painful.
- You notice **some pains in your pelvis** if you do anything strenuous.
- You get **tired and worn out** easily.
- You no longer have periods. However, you still have a regular hormonal cycle, without monthly bleeding.

Impact on your life

- You now have no risk of developing womb cancer.
- You do not need any more screening for womb cancer. You do not need to have cervical smear tests.
- You cannot get pregnant.
- You can do **most things to look after yourself, however you need help looking after your children**.
- You sometimes need to **rest throughout the day because you are tired**.
- **You have not yet gone back to work**. You have been told to avoid swimming, bathing, heavy lifting, and sexual intercourse for another 2 weeks.

[Scenario B]

About you

- You are 40 years old. You have children and have completed your family.
- You have no medical conditions affecting your general health.
- You have been told you carry a genetic alteration, which increases your risk of womb cancer in future.
- You have had yearly screening for womb cancer. This included an ultrasound scan and a small camera inserted into the womb to take a sample from the lining of the womb.
- You discussed your options to prevent womb cancer with a specialist. You decided to have an operation to remove your womb to prevent womb cancer.

Treatment

- **1 year** ago you had an operation to **remove your womb**. You did **not** have your ovaries removed.
- The operation went well, and you went home the next day.

Symptoms

- You have 4 small scars on your abdomen, each 1-2cm. These have fully healed.
- You have **no** pains from the operation.
- You no longer have periods. However, you still have a regular hormonal cycle, without monthly bleeding.

Impact on your life

- You now have no risk of developing womb cancer.
- You do not need any more screening for womb cancer. You do not need to have cervical smear tests.
- You cannot get pregnant.
- You have **no problems** looking after yourself or your children.
- Your sex life with your partner is the same as it was before surgery.

[Scenario C]

About you

- You are 40 years old. You have children and have completed your family.
- You have no medical conditions affecting your general health.
- You have been told you carry a genetic alteration, which increases your risk of womb cancer in future.
- You have had yearly screening for womb cancer. This included an ultrasound scan and a small camera inserted into the womb to take a sample from the lining of the womb.
- You discussed your options to prevent womb cancer with a specialist. You decided to have an operation to remove your womb to prevent womb cancer.

Treatment

- **1 month** ago you had an operation to remove your **womb including your ovaries**.
- The operation went well, and you went home the next day.
- You place a **hormonal skin patch** on your arm, which you change every few days.

Symptoms

- You have 4 small scars on your abdomen, each 1-2cm. These are healing well and are not painful.
- You notice some **pains in your pelvis** if you do anything strenuous.
- You get **tired and worn out** easily.
- You no longer have periods.
- Most days you have a **hot flush**, lasting a few minutes. These make you feel warm and sweaty. This can happen in the day or at night, and is unpredictable.
- You notice **some vaginal dryness**, which makes your vagina slightly more sensitive.

Impact on your life

- You now have no risk of developing womb cancer.
- You do not need any more screening for womb cancer. You do not need to have cervical smear tests.
- You cannot get pregnant.
- You can do **most things to look after yourself, however you need help looking after your children**.
- You sometimes need to **rest throughout the day because you are tired**.
- If a hot flush happens in the day, you may need to **stop what you are doing** for a few minutes, and take off an item of clothing to cool down.
- If a hot flush happens at night, it wakes you up and **disturbs your sleep**.
- **You have not yet gone back to work**. You have been told to avoid swimming, bathing, heavy lifting, and sexual intercourse for another 2 weeks.

[Scenario D]

About you

- You are 40 years old. You have children and have completed your family.
- You have no medical conditions affecting your general health.
- You have been told you carry a genetic alteration, which increases your risk of womb cancer in future.
- You have had yearly screening for womb cancer. This included an ultrasound scan and a small camera inserted into the womb to take a sample from the lining of the womb.
- You discussed your options to prevent womb cancer with a specialist. You decided to have an operation to remove your womb to prevent womb cancer.

Treatment

- **1 year** ago you had an operation to remove your **womb including your ovaries**.
- The operation went well, and you went home the next day.
- You place a **hormonal skin patch** on your arm, which you change every few days.

Symptoms

- You have 4 small scars on your abdomen, each 1-2cm. These have fully healed.
- You have **no** pains from the operation.
- You no longer have periods. However, you still have a regular hormonal cycle, without monthly bleeding.
- You feel slightly more **tired** than you did before surgery. You can be more **forgetful**.
- Provided that you change your skin patches regularly, you do **not** notice any hot flushes in the day or night. If you forget to change these then you do experience hot flushes.

Impact on your life

- You now have no risk of developing womb cancer.
- You do not need any more screening for womb cancer. You do not need to have cervical smear tests.
- You cannot get pregnant.
- You have **no problems** looking after yourself or your children.
- You can be **less interested in sex** with your partner than you were before surgery. When you have sex you sometimes feel slight discomfort and need to use **lubrication**.

Health of the person described

Please imagine that you are the person described in the scenario

Please rate how good or bad “your” health is, based on the description.

This scale is numbered from 0 to 100.

100 means the <u>best</u> health you can imagine.
0 means the <u>worst</u> health you can imagine.

Please **mark an X** on the scale to indicate how “your" health is TODAY.

Now, write the number you marked on the scale in the box below.

YOUR HEALTH = [ ]

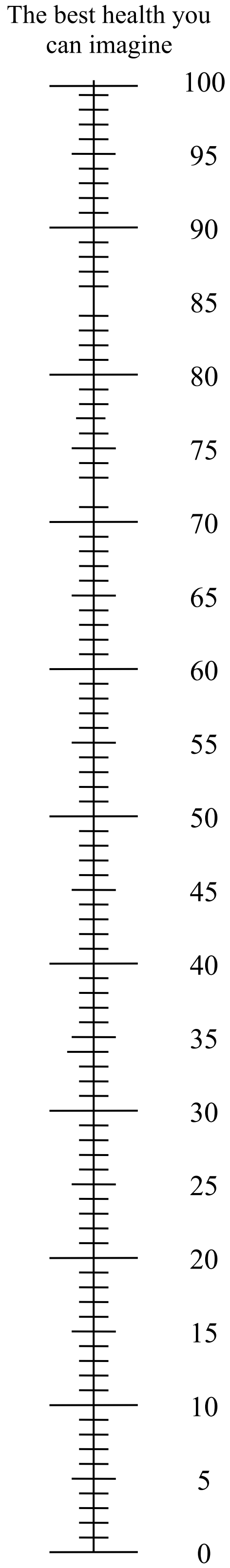

Quality of life of the person described

Please imagine that you are the person described in the scenario

Under each heading, please tick the ONE box that best describes "your" health.

MOBILITY

I have no problems in walking about ❑

I have slight problems in walking about ❑

I have moderate problems in walking about ❑

I have severe problems in walking about ❑

I am unable to walk about ❑

SELF-CARE

I have no problems washing or dressing myself ❑

I have slight problems washing or dressing myself ❑

I have moderate problems washing or dressing myself ❑

I have severe problems washing or dressing myself ❑

I am unable to wash or dress myself ❑

USUAL ACTIVITIES *(e.g. work, study, housework, family or leisure activities)*

I have no problems doing my usual activities ❑

I have slight problems doing my usual activities ❑

I have moderate problems doing my usual activities ❑

I have severe problems doing my usual activities ❑

I am unable to do my usual activities ❑

PAIN / DISCOMFORT

I have no pain or discomfort ❑

I have slight pain or discomfort ❑

I have moderate pain or discomfort ❑

I have severe pain or discomfort ❑

I have extreme pain or discomfort ❑

ANXIETY / DEPRESSION

I am not anxious or depressed ❑

I am slightly anxious or depressed ❑

I am moderately anxious or depressed ❑

I am severely anxious or depressed ❑

I am extremely anxious or depressed ❑

Section 3: Your own health and demographics

We would now like to ask a few more questions about your own health. This relates to YOU and not the person in the scenario. These can be important in understanding a person's quality-of-life.

1. Do you have any of these medical conditions (Please select all that apply)
    a. Obesity (BMI 30 or more)
    b. Diabetes
    c. Polycystic ovarian syndrome
    d. Heavy periods
    e. Endometriosis
    f. High blood pressure

2. Have you ever been diagnosed with cancer? (Please select one)
    a. Yes
    b. No
    c. Not sure

    If yes, what type of cancer? ______________
    [*Options – breast, lung, bowel, womb, melanoma skin, ovary, brain, lymphoma, pancreas, kidney, other]*

3. Do you have a family history of womb, ovarian or cervical cancer?
    a. Yes
    b. No
    c. Not sure

4. Which statement best describes your periods? (Please select one)
    a. I still have regular periods
    b. My periods are irregular, but I have had at least 1 period in the last 12 months
    c. I have not had a period at all in the last 12 months

5. If you are not having regular periods, what is the reason? (Please select all applicable)
    a. Natural menopause
    b. Mirena coil/ implant/ pill/ other contraception
    c. Surgery (for example, removal of womb)
    d. Chemotherapy
    e. Radiation treatment
    f. Don't know
    g. Other (please describe the reason) - *[free text box]*

6. Do you have children? (Please select one)
    a. Yes
    b. No

7. Do you plan to have (more) children in the future? (Please select one)
    a. Yes

b. No
c. Not sure

The next questions are about your personal circumstances, which can be important in understanding quality-of-life. These are optional, please complete if you feel able to.

8. How would you describe your ethnic background? (Please select one)
    a. White
    b. Black
    c. Asian
    d. Mixed
    e. Other (please describe below) - *[free text box]*

9. What is your relationship status? (Please select one)
    a. Single
    b. In a relationship
    c. Married/ civil partnership/ living with a partner
    d. Separated/ divorced/ widowed

10. What is your highest level of educational qualification? (Please select one)
    a. No formal qualifications
    b. GCSE/ O-level or equivalent
    c. A-Level or equivalent
    d. Undergraduate degree or equivalent
    e. Postgraduate degree or equivalent
    f. Other (please describe) - *[free text box]*

11. Of the following income groups, which best represents your total household income in the last 12 months? (Please select one)
    a. Less than £10,000
    b. £10,000 - £19,999
    c. £20,000 - £29,999
    d. £30,000 - £39,999
    e. £40,000 - £49,999
    f. £50,000 or more

12. What is the first half of your postcode? _______

Thank you for completing the questionnaire

We really appreciate you taking the time to complete this study. Your participation will help us develop better strategies to prevent womb cancer in future.

What if I have further questions about this study?

Please do not hesitate to contact us:

Email: [contact email address]

Or write to: [contact post address]

Further information on womb cancer can be found at:

Lynch Syndrome UK
https://www.lynch-syndrome-uk.org/
Lynch Syndrome UK are a charity which supports and has helped develop this study

NHS or **Cancer Research UK:**
https://www.nhs.uk/conditions/womb-cancer/
https://www.cancerresearchuk.org/about-cancer/womb-cancer

If you have any immediate health concerns arising from this study, please speak to your GP.
