# Supplementary material for: Health-related quality of life after risk-reducing hysterectomy (PRESCORES): a cross-sectional study
Source: eClinicalMedicine. 2026 Jul 27;98:104083. doi: 10.1016/j.eclinm.2026.104083 (PMC13523832; doi:10.1016/j.eclinm.2026.104083)
Supplement: Supplementary file 2 [file mmc2.docx]

**Figure S1:**	Locations of PRESCORES recruiting sites within the UK

**Table S1**:	Number (percentage) of participants reporting each EQ-5D domain.

|  | RRH | No-RRH | Total | P-value |
| --- | --- | --- | --- | --- |
| n | 529 | 591 | 1,120 |  |
| Mobility | | | |  |
| No problems | 404 (76.4) | 511 (86.5) | 915 (81.7) | <0.0001* |
| Slight problems | 71 (13.4) | 46 (7.8) | 117 (10.5) |  |
| Moderate problems | 34 (6.4) | 29 (4.1) | 63 (5.6) |  |
| Severe problems | 18 (3.4) | 4 (0.7) | 22 (2.0) |  |
| Unable to | 2 (0.4) | 1 (0.2) | 3 (0.3) |  |
| Self-care | | | |  |
| No problems | 482 (91.1) | 558 (94.3) | 1,040 (92.9) | 0.20 |
| Slight problems | 27 (5.1) | 19 (3.2) | 46 (4.1) |  |
| Moderate problems | 16 (3.0) | 11 (1.9) | 27 (2.4) |  |
| Severe problems | 4 (0.8) | 3 (0.5) | 7 (0.6) |  |
| Unable to | 0 | 0 | 0 |  |
| Usual activities | | | |  |
| No problems | 388 (73.4) | 489 (82.7) | 877 (78.3) | 0.0010* |
| Slight problems | 87 (16.5) | 59 (10.0) | 146 (13.0) |  |
| Moderate problems | 34 (6.4) | 35 (5.9) | 69 (6.2) |  |
| Severe problems | 17 (3.2) | 7 (1.2) | 24 (2.1) |  |
| Unable to | 3 (0.6) | 1 (0.2) | 4 (0.4) |  |
| Pain/discomfort | | | |  |
| None | 278 (52.6) | 359 (60.7) | 637 (56.9) | 0.013* |
| Slight | 170 (32.1) | 164 (27.8) | 334 (29.8) |  |
| Moderate | 46 (8.7) | 50 (8.5) | 96 (8.6) |  |
| Severe | 26 (4.9) | 14 (2.4) | 40 (3.6) |  |
| Extreme | 9 (1.7) | 4 (0.7) | 13 (1.2) |  |
| Anxiety/depression | | | |  |
| None | 267 (50.5) | 268 (45.4) | 535 (47.8) | 0.52 |
| Slight | 162 (30.6) | 195 (33.0) | 357 (31.9) |  |
| Moderate | 77 (14.6) | 101 (17.1) | 178 (15.9) |  |
| Severe | 17 (3.2) | 19 (3.2) | 36 (3.2) |  |
| Extreme | 6 (1.1) | 8 (1.4) | 14 (1.3) |  |

RRH – risk-reducing hysterectomy; P-value indicates the result of a Mann-Whitey U test to compare RRH and no-RRH groups	* Indicates statistical significance at 0.05.

**Table S2:**	Current unadjusted utilities and VAS within RRH and no-RRH groups, including separation of RRH group by current age (<51 vs ≥51years), and separation of no-RRH group by menopausal status (pre- vs post-menopausal).

|  | RRH  (all) | No-RRH (all) | RRH (<51yrs) | No-RRH (Premen) | RRH (≥51yrs) | No-RRH (Postmen) | Overall |
| --- | --- | --- | --- | --- | --- | --- | --- |
| Age, years mean [range] | 54.9  [32- 82] | 42.9  [18 - 87] | 44.5  [32-50] | 36.2  [18-55] | 60.7  [51-82] | 61.1  [26-87] | 48.6  [18 - 87] |
| *EQ-5D utilities* | | | | | | | |
| N | 529 | 591 | 189 | 433 | 340 | 158 | 1,120 |
| Mean | 0.793 | 0.828 | 0.796 | 0.847 | 0.792 | 0.778 | 0.812 |
| Median | 0.858 | 0.871 | 0.817 | 0.890 | 0.858 | 0.851 | 0.860 |
| SD | 0.225 | 0.189 | 0.188 | 0.164 | 0.243 | 0.237 | 0.207 |
| 25^th^ | 0.741 | 0.786 | 0.738 | 0.797 | 0.742 | 0.700 | 0.757 |
| 75^th^ | 0.985 | 0.985 | 0.890 | 0.985 | 0.986 | 0.985 | 0.985 |
| Minimum | -0.278 | -0.166 | -0.032 | -0.001 | -0.278 | -0.166 | -0.278 |
| Maximum | 0.988 | 0.988 | 0.985 | 0.986 | 0.988 | 0.988 | 0.988 |
| *VAS* |  |  |  |  |  |  |  |
| N | 523 | 589 | 189 | 431 | 334 | 158 | 1,112 |
| Mean | 78.0 | 78.8 | 75.8 | 80.2 | 79.3 | 74.9 | 78.4 |
| Median | 80.0 | 80.0 | 80.00 | 84.0 | 85.0 | 80.0 | 80.0 |
| SD | 17.6 | 15.8 | 17.1 | 14.9 | 17.7 | 17.6 | 16.7 |
| 25^th^ | 70.0 | 72.0 | 70.0 | 75.0 | 73.0 | 69.0 | 70.0 |
| 75^th^ | 90.0 | 90.0 | 88.0 | 90.0 | 90.0 | 90.0 | 90.0 |
| Minimum | 6.0 | 7.0 | 10.0 | 7.0 | 6.0 | 20.0 | 6.0 |
| Maximum | 100.0 | 100.0 | 100.0 | 100.0 | 100 | 100.0 | 100.0 |

RRH – risk-reducing hysterectomy; Premen – premenopausal; Postmen – postmenopausal; SD – standard deviation; VAS – visual analogue scale; 25^th^ – 25^th^ percentile, 75^th^ – 75^th^ percentile

**Table S3**:	Sensitivity analysis of primary analysis using participants aged 35-80 years.

| **Impute** | **Model** | **Overall effect (95%CI)** | **Pre-menopausal effect (95%CI)** | **Post-menopausal effect (95%CI)** |
| --- | --- | --- | --- | --- |
| No | Regression | -0.0131  (-0.0383 to 0.0130) | -0.0227  (-0.0555 to 0.0080) | -0.0048  (-0.0382 to 0.0293) |
| Yes | Regression | -0.0129  (-0.0414 to 0.0127) | -0.0234  (-0.0554 to 0.0069) | -0.0038  (-0.0419 to 0.0287) |
| No | PS+IPW | -0.0101  (-0.0383 to 0.0181) | -0.0421  (-0.0758 to -0.0084) | 0.0179  (-0.0255 to 0.0613) |
| Yes | PS+IPW | -0.0130  (-0.0376 to 0.0116) | -0.0318  (-0.0630 to -0.0006) | 0.0035  (-0.0357 to 0.0427) |
| No | PS+IPW (DR) | -0.0119  (-0.0383 to 0.0154) | -0.0339  (-0.0662 to -0.0034) | -0.0001  (-0.0379 to 0.0376) |
| Yes | PS+IPW (DR) | -0.0133  (-0.0392 to 0.0134) | -0.0310  (-0.0628 to 0.0001) | 0.0024  (-0.0360 to 0.0400) |

Model: Regression: Linear regression, adjusted for same factors as primary analysis; PS: Propensity score; IPW: inverse probability weighting; DR: doubly-robust estimate; Impute: multiple imputation (Yes) or complete-case analysis (No).

**Table S4**:	Sensitivity analysis of RRH without BSO vs all no-RRH, including all patients overall, and separating RRH group by current age (</≥51years), and no-RRH group by menopausal status (pre/post-menopausal).

|  | n RRH/no-RRH | Adjusted mean difference in utilities | Bootstrap SE | 95% CI | *P-*value |
| --- | --- | --- | --- | --- | --- |
| *Sensitivity analysis of RRH without BSO vs no-RRH* | | | | | |
| All RRH vs all no-RRH | 24/554 | -0.111^a^ | 0.059 | -0.227, -0.005 | 0.061 |
| Premen-age RRH vs premen no-RRH | 15/413 | -0.013^a^ | 0.047 | -0.106, 0.080 | 0.78 |
| Postmen-age RRH vs postmen no-RRH | 9/141 | -0.284^a^ | 0.110 | -0.498, -0.070 | 0.092 |

RRH – risk-reducing hysterectomy; SE – standard error; CI – confidence interval; Premen – premenopausal; Postmen – postmenopausal

^a^adjusted for physical health, mental health, income, cancer history and age

**Table S5:**	Unadjusted mean difference in EQ-5D utilities between RRH and no-RRH groups, stratified by time since surgery in the RRH group.

|  |  | n RRH/no-RRH (total) | Mean difference in utilities [SE] | 95% CI | *P-value* |
| --- | --- | --- | --- | --- | --- |
| RRH (<51years) vs no-RRH (premenopausal) | 0 – 2 years | 86/433 (519) | -0.049 [0.019] | -0.086, -0.013 | 0.0075 |
|  | 3 – 5 years | 40/433 (473) | -0.041 [0.026] | -0.093, 0.010 | 0.12 |
|  | ≥6 years | 56/433 (489) | -0.070 [0.034] | -0.137, -0.002 | 0.043 |
| RRH (≥51years) vs no-RRH (postmenopausal) | 0 – 2 years | 61/158 (219) | 0.041 [0.034] | -0.026, 0.108 | 0.23 |
|  | 3 – 5 years | 54/158 (212) | 0.013 [0.035] | -0.055, 0.082 | 0.67 |
|  | ≥6 years | 205/158 (363) | 0.004 [0.026] | -0.047, 0.056 | 0.87 |

RRH – risk-reducing hysterectomy; SE – standard error; CI – confidence interval

**Table S6:**	Adjusted mean difference in retrospective utilities between RRH and no-RRH groups, overall, and by age at RRH (</≥51years), and menopausal status for the no-RRH group.

|  | n RRH/ no-RRH | Adjusted mean difference in utilities | Bootstrap SE | 95% CI | *P-value* |
| --- | --- | --- | --- | --- | --- |
| RRH vs no-RRH^a^ | 454/554 | -0.026 | 0.014 | -0.054, 0.001 | 0.063 |
| RRH (<51years) vs no-RRH (premenopausal)^a^ | 288/413 | -0.080 | 0.019 | -0.118, -0.043 | <0.001 |
| RRH (≥51 years) vs no-RRH (postmenopausal)^a^ | 166/141 | 0.036 | 0.023 | -0.009, 0.081 | 0.11 |

RRH – risk-reducing hysterectomy; SE – standard error; CI – confidence interval

^a^adjusted for physical health, mental health, income, cancer history and age

**Table S7:**	Cancer Worry Scale scores for RRH & no-RRH groups, and overall

| Cancer Worry Scale  (total score) | RRH | No-RRH | Overall | *P*-value |
| --- | --- | --- | --- | --- |
| N | 521 | 582 | 1103 |  |
| Mean | 7.8 | 8.3 | 8.1 | 0.0037* |
| Median | 7.0 | 8.0 | 8.0 |  |
| SD | 2.7 | 2.8 | 2.8 |  |
| 25^th^ | 6.0 | 6.0 | 6.0 |  |
| 75^th^ | 10.0 | 10.0 | 10.0 |  |

SD – standard deviation; 25th – 25th percentile, 75th – 75th percentile

**Figure S2:**	Cancer Worry Scale and unadjusted EQ-5D utility scores, for all patients and RRH and no-RRH groups.

All women

RRH group

no-RRH group

**Table S8:**	Utilities of the study population (overall and with/without a personal cancer history), unadjusted and adjusted by age-standardised using against the reference female general population in England*^33^* (shown in the last row).

| Age, years  N (%) | 18-24  22  (2.0) | 25-29  76 (6.8) | 30-34  96 (8.6) | 35-39  141 (12.6) | 40-44  122 (10.9) | 45-49  134 (12.0) | 50-54  126 (11.3) | 55-59  137 (12.3) | 60-64  120 (6.7) | 65-69  75 (6.7) | 70-74  40 (3.6) | 75+  28  (2.5) | Overall  1,117 (100) |
| --- | --- | --- | --- | --- | --- | --- | --- | --- | --- | --- | --- | --- | --- |
| *All study participants* | | | | | | | | | | | | | |
| Utilities (unadjusted) | 0.792 | 0.849 | 0.853 | 0.836 | 0.841 | 0.811 | 0.801 | 0.805 | 0.761 | 0.784 | 0.773 | 0.791 | 0.812 |
| Utilities (Age-standardised) |  |  |  |  |  |  |  |  |  |  |  |  | 0.808 |
| *Participants with a personal cancer history* | | | | | | | | | | | | | |
| Utilities (unadjusted) | - | 0.855 | 0.869 | 0.796 | 0.816 | 0.803 | 0.828 | 0.807 | 0.731 | 0.772 | 0.776 | 0.803 | 0.788 |
| Utilities (Age-standardised) |  |  |  |  |  |  |  |  |  |  |  |  | 0.805 |
| *Participants without a personal cancer history* | | | | | | | | | | | | | |
| Utilities (unadjusted) | 0.792 | 0.848 | 0.851 | 0.841 | 0.850 | 0.815 | 0.789 | 0.802 | 0.806 | 0.796 | 0.766 | 0.755 | 0.823 |
| Utilities (Age-standardised) |  |  |  |  |  |  |  |  |  |  |  |  | 0.808 |
| *Reference female general population in England^33^* | | | | | | | | | | | | | |
| N  (% of total) | 492  (6.2) | 515 (6.5) | 669 (8.4) | 722 (9.1) | 668 (8.4) | 693 (8.7) | 729 (9.2) | 730 (9.2) | 608 (7.7) | 619 (7.8) | 619 (7.8) | 879 (11.1) | 7943  (100) |
| Utilities | 0.858 | 0.869 | 0.869 | 0.854 | 0.846 | 0.806 | 0.798 | 0.791 | 0.776 | 0.775 | 0.784 | 0.708 | 0.808 |

Utilities are provided for the PRESCORES study population, overall, and for those subset of participants with and without a personal cancer history, with the reference female general population provided at the bottom. Utilities are provided for each participant age group, with overall (mean) values on the right. For PRESCORES participants, utilities are provided unadjusted, and adjusted by age-standardisation against the reference female general population. Utilities from the PRESCORES study were age-standardised to the 2023 McNamara population.(1) To calculate the age-standardised utilities, the age-specific PRESCORES utilities were multiplied by the proportion of the McNamara population belonging to the particular age group, for each age group, and then added together
